# Serum Concentrations of Trace Elements Zinc, Copper, Selenium, and Manganese in Critically Ill Patients

**DOI:** 10.1007/s12011-018-1429-4

**Published:** 2018-07-25

**Authors:** Yeon Hee Lee, Eun-Sook Bang, Ji-Hyun Lee, Jung-Dong Lee, Dae Ryong Kang, Jeong Hong, Jae-Myeong Lee

**Affiliations:** 1 Food Service and Clinical Nutrition Team, Ajou University Hospital, Worldcup-ro 164, Yeongtong-gu, Suwon, 16499 South Korea; 2 Department of Pharmacy, Ajou University Hospital, Worldcup-ro 164, Yeongtong-gu, Suwon, 16499 South Korea; 3 Office of Biostatistics, Ajou University School of Medicine, Worldcup-ro 164, Yeongtong-gu, Suwon, 16499 South Korea; 4 Department of Surgery, Ajou University School of Medicine, Worldcup-ro 164, Yeongtong-gu, Suwon, 16499 South Korea; 5 Department of Acute Care Surgery, Department of Surgery, Korea University Anam Hospital, Inchon-ro 73, Seongbuk-gu, Seoul, South Korea

**Keywords:** Critically ill patient, Trace element, Zinc, Copper, Selenium, Manganese

## Abstract

We measured serum concentrations of trace elements and evaluated their clinical significance in relation to treatment outcomes of critically ill patients. A total of 167 participants (105 men and 62 women; average age, 61.4 years; age range, 18–90 years) were enrolled. Arterial blood concentrations of the trace elements zinc, copper, selenium, and manganese were measured every 14 days. At the time of intensive care unit (ICU) admission, serum concentrations of zinc, selenium, copper, and manganese were lower than the normal values in 75.1, 1.8, 37.8, and 2.1% of patients, respectively. Serum trace element concentrations measured on day 14 of ICU stay were higher than those measured at the time of ICU admission for zinc (53.3 → 80.7 μg/L) and copper (87.1 → 102.3 μg/L). Increased serum zinc and copper concentrations during ICU care were associated with a significantly lower mortality compared to decreased concentrations of zinc (15.6 vs. 83.3%, *p* = 0.003) and copper (5.6 vs. 50.0%, *p* = 0.013). At the time of ICU admission, low serum levels of zinc and copper were observed. Patients with increased serum concentrations of zinc and copper had significantly lower mortality.

## Introduction

Severe diseases are characterized by inflammation, oxidative stress, and immune failure. Inflammatory reactions may lead to the loss of intestinal mucosal integrity, which may decrease the absorption of essential nutrients and increase oxidative stress, resulting in a worsened condition [1]. In severely ill patients, the network of antioxidant defense mechanisms (e.g., superoxide dismutase, catalase, and glutathione peroxidase) formed by trace element-dependent enzymes may protect cells from reactive oxygen species and nitric oxide [2]. Trace elements such as zinc (Zn), selenium (Se), manganese (Mn), and copper (Cu) protect cells from oxidative stress [1].

The biological functions of Zn include the maintenance of normal growth, immune function, DNA repair, protein synthesis, glucose control, and wound healing [3]. Zn deficiency has been reported in critically ill patients with septic shock [4]. Se is an essential trace element with antioxidant, immunological, and anti-inflammatory properties. Cu is a component of the Cu/Zn superoxide dismutase and serves as a free radical scavenger [1]. Finally, Mn is also an essential part of superoxide dismutase that reduces the effect of oxidative stress on mitochondria [1].

Blood concentrations of trace elements decrease after severe trauma, surgery, sepsis, and severe systemic inflammatory response [1, 5]. Thereafter, they are maintained at low levels for several days or weeks [6]. Low levels of trace elements in critically ill patients may reflect malnutrition. However, studies of the effects of trace element deficiencies on mortality of critically ill patients and the length of stay in the hospital and intensive care unit (ICU) are inconsistent. The recently published American Society of Parenteral and Enteral Nutrition (ASPEN) guidelines and Canadian guidelines do not recommend Se, Zn, and antioxidant supplementation in patients with sepsis due to conflicting data [5]. Conversely, the European Society of Parenteral and Enteral Nutrition guidelines recommend daily supplementation with multivitamins and trace elements for all parenteral nutrition (PN) prescriptions for ICU patients [7].

In this study, we measured the concentrations of trace elements in blood samples of surgical ICU patients and evaluated their clinical significance.

## Materials and Methods

This was a prospective and observational study. The research participants included 167 patients selected among those admitted to the surgical ICU of Ajou University Hospital between December 2014 and November 2015 and who received input from the nutrition support team. Age, sex, mortality, severity indices for critically ill patients, length of stay in the ICU, and length of stay in the hospital were recorded. We used Simplified Acute Physiology Score (SAPS) III, Sequential Organ Failure Assessment (SOFA) scores, and Acute Physiology and Chronic Health Evaluation (APACHE) II scores to determine the severity indices for critically ill patients from patients’ clinical data obtained within 24 h of admission. Their malnutrition status was evaluated based on the International Classification of Diseases, 9th revision, Clinical Modification.

Zn, Cu, Se, and Mn concentrations were measured in arterial blood sampled every 14 days starting from the time of ICU admission until patient discharge from the ICU. Serum concentrations of Zn, Cu, Se, and Mn were measured by an external testing agency (Greencross Laboratory, Yong-in City, South Korea). Serum concentrations of Zn and Cu were analyzed using inductively coupled plasma-mass spectroscopy, while Se and Mn were analyzed using atomic absorption spectroscopy. Five days was required to process the test results. Serum concentrations were measured every 14 days until the patient was discharged from the ICU. The reference range for each trace element was as follows: Zn (66–110 μg/dL), Se (5.8–23.4 μg/dL), Cu (75–145 μg/dL), and Mn (4.7–18.3 μg/dL) (http://www.gclabs.co.kr/eng/information/search_test_item_view).

The patients received various nutrition therapies (e.g., total intravenous alimentation, intravenous alimentation and enteral nutrition, and total enteral nutrition) depending on their clinical status. A daily supplementary injection was provided to all patients using a standard formulation of trace elements (Cu 1 mg, Mn 50 μg, Zn 5.0 mg, Chromium 10 μg). However, no separate Se supplementation was provided.

IBM SPSS Statistics for Windows, version 23.0 (IBM Corp., Armonk, NY, USA), was used for statistical analyses. Data are shown as means (± standard deviation, SD, with range) for continuous variables or numbers and as percentages for categorical variables. Comparisons between subgroups according to patient age, mortality, and serum trace element values were tested for significance by unpaired Student’s *t* tests for continuous variables and Pearson chi-square tests for categorical variables. Patient survival analysis was performed using the Kaplan-Meier method. *P* values of < 0.05 were considered statistically significant.

## Results

**Fig. 1:**
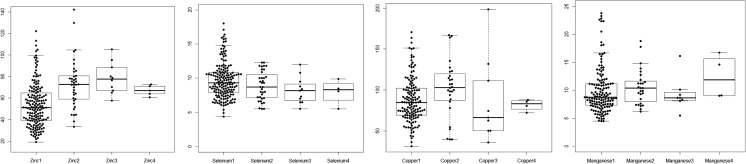
Serum concentration of trace elements (zinc [Zn], selenium [Se], copper [Cu], and manganese [Mn]) tested every 2 weeks during the intensive care unit (ICU) stay. Zn unit and normal range 66–110 μg/dL, Cu unit and normal range 75–145 μg/dL, Mn unit and normal range 4.7–18.3 μg/dL, Se unit and normal range 5.8–23.4 μg/dL

During the research period, 167 patients comprising 105 men (62.9%) and 62 women (37.1%) were registered. Their average age was 61.4 ± 14.9 years (range 18–90 years). Of these, 29 (17.3%) ICU patients died during the study period. The average APACHE II, SOFA, and SAPS III scores were 18.2 ± 7.3, 6.7 ± 3.7, and 45.1 ± 16.9, respectively. The average ICU length of stay was 13.1 days, while the average hospital length of stay was 42.3 days. Nutrition evaluation conducted after ICU admission revealed evidence of malnutrition in 116 patients (69.5%). The average concentrations of Zn, Se, Cu, and Mn in the serum samples of the 167 patients at ICU admission were 53.4 ± 50.4, 9.8 ± 2.6, 87.1 ± 28.3, and 10.0 ± 4.2 μg/dL, respectively (Table 1, Fig. 1). At the time of ICU admission, Zn, Se, Cu, or Mn deficiency was present in 127 (76%), three (1.8%), 54 (37.8%), and three (2.1%) patients, respectively.

**Table 1:** Serum trace element concentrations during the ICU stay

	Serum concentration	Zinc (μg/dL)	Selenium (μg/dL)	Copper (μg/dL)	Manganese (μg/dL)
	Reference range	66–110	5.8–23.4	75–145	4.7–18.3
Day 1	*N*	167	167	140	140
	Mean value ± SD, μg/dL	53.4 ± 20.4	9.8 ± 2.6	87.1 ± 28.3	10.0 ± 4.2
	Range, μg/dL	19.2–122.1	4.4–18.0	11.3–170.5	4.5–23.9
	*n* (% below reference range)	127 (75.1)	3 (1.8)	54 (37.8)	3 (2.1)
	*n* (% above reference range)	2 (1.2)	0 (0)	6 (4.2)	8 (5.6)
Day 15	*N*	38	35	28	27
	Mean value ± SD, μg/dL	72.8 ± 22.6	9.0 ± 2.1	102.3 ± 34.1	10.6 ± 3.2
	Range, μg/dL	33.4–142	5.5–12.3	39.5–166.8	6.2–18.8
Day 29	*N*	10	10	7	6
	Mean value ± SD, μg/dL	78.4 ± 14.6	8.7 ± 2.2	95.1 ± 56.7	9.6 ± 3.5
	Range, μg/dL	57.8–105.1	5.5–12.0	36.1–198.6	5.5–16.1
Day 43	*N*	6	5	5	5
	Mean value ± SD, μg/dL	69.4 ± 5.0	8.0 ± 1.6	78.6 ± 9.1	11.5 ± 3.8
	Range, μg/dL	60.6–74.1	5.5–9.9	66.5–88.0	8.3–16.7

*ICU*, intensive care unit; *SD*, standard deviation

Comparison of survivors and non-survivors (Table 2) revealed a significant difference in SOFA and SAPS III scores. However, the APACHE II score was not different. The average ICU length of stay for non-survivors (19.1 days) was significantly longer than that for survivors (11.8 days) (*P* = 0.017), whereas the average hospital length of stay for survivors (42.2 days) was significantly longer than that for non-survivors (29.9 days) (*P* = 0.039). The number of malnourished non-survivors was significantly greater than that of malnourished survivors (*P* = 0.009). No significant differences in the concentrations of Zn, Se, Cu, and Mn were evident in the serum obtained from survivors and non-survivors at the time of ICU admission. In this study, 70 subjects were older than 65 years and there were more male than female patients; however, there were no statistically significant differences in age and sex between these groups (Tables 3 and 4).

**Table 2:** Demographic characteristics of the patients at ICU admission (*n* = 167)

Variable	Total (*n* = 167)	Survivors (*n* = 138)	Non-survivors (*n* = 29)	*P* value
Age, years (range)	61.4 ± 14.9 (18–90)	61.3 ± 14.8	61.9 ± 14.0	0.848
Sex (male), *n* (%)	105 (62.9)	89 (64.5)	16 (55.2)	0.400
APACHE II	18.2 ± 7.3	17.7 ± 7.2	19.9 ± 8.1	0.151
SOFA	6.7 ± 3.7	6.1 ± 3.3	9.3 ± 4.7	0.001
SAPS III	45.1 ± 16.9	43.0 ± 16.0	53.5 ± 20.3	0.003
ICU stay (days)	13.1 ± 15.0	11.8 ± 14.5	19.1 ± 16.2	0.017
Hospital stay (days)	42.3 ± 38.5	42.2 ± 30.1	29.9 ± 22.9	0.039
Ventilator (days; *n* = 76)	16.1 ± 44.3	10.6 ± 14.7 (*n* = 59)	34.4 ± 88.6 (*n* = 17)	0.286
CRRT (days; *n* = 19)	10.7 ± 8.9	12.6 ± 12.7 (*n* = 7)	9.7 ± 6.1 (*n* = 12)	0.506
Sepsis (%)	138 (82.6)	96 (70.0)	17 (58.6)	0.279
Septic shock (%)	73 (43.7)	45 (32.6)	28 (96.6)	0.000
Trace elements, μg/dL				
Zinc	53.42 ± 20.4	52.49 ± 19.6	57.83 ± 23.8	0.201
Selenium	9.75 ± 2.6	9.76 ± 2.7	9.35 ± 2.3	0.439
Copper	87.09 ± 28.3	86.02 ± 27.6	92.40 ± 29.3	0.335
Manganese	9.96 ± 4.2	9.88 ± 4.0	10.2 ± 4.9	0.735
Nutrition status, *n* (%)				0.009
Well nourished	51 (30.5)	48 (34.8)	3 (10.3)	
Malnourished	116 (69.5)	90 (65.2)	26 (89.7)	.
Diagnostic group, *n* (%)				0.160
Gastrointestinal	49 (29.3)	41	8	
Hepatopancreatobiliary	55 (32.9)	41	14	
Cardiovascular	9 (5.4)	7	2	
Miscellaneous	54 (32.3)	49	5	

*APACHE*, Acute Physiology and Chronic Health Evaluation; *SOFA*, Sequential Organ Failure Assessment; *SAPS*, Simplified Acute Physiology Score; *ICU*, intensive care unit; *CRRT*, continuous renal replacement therapy

**Table 3:** Serum trace element concentrations by age group

Variable	Age (years)		*P* value
	< 65 (*N* = 97)	≥ 65 (*N* = 70)	
Sex (male), *n* (%)	62 (63.9)	43 (61.4)	
Trace elements, μg/dL			
Zinc	53.2 ± 20.4	53.7 ± 20.5	0.884
Selenium	9.8 ± 2.5	9.6 ± 2.7	0.574
Copper	86.6 ± 29.7	88.2 ± 25.8	0.732
Manganese	10.3 ± 4.5	9.4 ± 3.4	0.193

**Table 4:** Serum trace element concentrations by sex

Variable	Sex		*P* value
	Male (*N* = 105)	Female (*N* = 62)	
Age, years (range)	61.0 ± 1.3 (18–88)	61.6 ± 1.9 (23–90)	0.744
Trace elements, μg/dL			
Zinc	54.22 ± 21.3	52.12 ± 18.9	0.516
Selenium	9.82 ± 2.5	9.52 ± 2.7	0.433
Copper	89.52 ± 28.1	83.02 ± 28.0	0.187
Manganese	9.62 ± 4.2	10.52 ± 4.0	0.258

We compared patients with and without low serum Zn and Cu levels at the time of ICU admission. Patients with sepsis or septic shock did not show significantly lower levels of the four trace elements. Those with low serum levels of Zn had higher severity scores than those without low serum levels of Zn, but the difference was not statistically significant (Table 5). Those with low serum levels of Zn had longer periods of ventilator use, although the difference was also not statistically significant. However, the group with normal Zn level had a significantly longer total duration of stay as compared to that in the low serum level group (52.8 vs. 38.9 days, *P* = 0.012). In addition, a significant number of patients with low serum levels of Zn also had low serum levels of Cu (*P* = 0.003). Regarding severity scores, patients with low levels of Cu had a higher value than that in the group of patients with normal values and the severity scores were significantly correlated with the APACHE II and SOFA scores *(P =* 0.043 and *P* = 0.037, respectively; Table 6). Among patients with septic shock, the serum Cu concentration was significantly lower (*P* = 0.045). In addition, patients with low serum levels of Cu tended to have low Zn and Se concentrations, with a statistically significant correlation with low levels of Se (*P* = 0.037).

**Table 5:** Characteristics of patients with low serum zinc level

Variable	Low level (*n* = 127)	Normal level (*n* = 40)	*P* value
Age, years (range)	61.41 (23–90)	61.33 (18–83)	0.990
Sex (male), *n* (%)	78 (61.4)	27 (67.5)	0.649
APACHE II	18.0 ± 7.4	18.6 ± 7.1	0.860
SOFA	6.5 ± 3.6	7.3 ± 4.1	0.319
SAPS III	44.7 ± 16.9	46.2 ± 17.3	0.893
Mortality, *n* (%)	21 (16.5)	8 (20.0)	0.678
ICU stay (days)	12.0 ± 13.6	16.5 ± 18.7	0.064
Hospital stay (days)	38.9 ± 38.2	52.8 ± 38.1	0.012
Ventilator (days)	16.6 ± 47.4	12.6 ± 14.6	0.726
CRRT (days)	8.4 ± 5.2	17.4 ± 13.8	0.110
Sepsis	38 (29.9)	15 (37.5)	0.315
Septic shock (%)	54 (42.5)	19 (47.5)	0.832
Trace elements, μg/dL			
Zinc	44.4 ± 11.7	82.1 ± 14.9	0.000
Selenium	9.8 ± 2.6	9.5 ± 2.7	0.508
Copper	82.8 ± 25.4	101.7 ± 32.9	0.003
Manganese	9.97 ± 4.4	9.94 ± 3.4	0.868
Nutrition status, *n* (%)			0.839
Well nourished	39 (30.7)	12 (30.0)	
Malnourished	88 (69.3)	28 (70.0)	

*APACHE*, Acute Physiology and Chronic Health Evaluation; *SOFA*, Sequential Organ Failure Assessment; *SAPS*, Simplified Acute Physiology Score; *ICU*, intensive care unit; *CRRT*, continuous renal replacement therapy

**Table 6:** Characteristics of patients with low serum copper level

Variable	Low level (*n* = 54)	Normal level (*n* = 86)	*P* value
Age (years)	59.7 ± 14.8	60.7 ± 14.2	0.778
Sex (male), *n* (%)	33 (61.1)	14 (16.3)	0.538
APACHE II	19.1 ± 8.0	16.9 ± 6.8	0.043
SOFA	7.4 ± 4.5	6.1 ± 3.2	0.037
SAPS III	47.3 ± 21.1	42.0 ± 13.8	0.057
Mortality, *n* (%)	9 (16.6)	12 (14.0)	0.962
ICU stay (days)	10.8 ± 14.4	12.0 ± 12.3	0.619
Hospital stay (days)	39.2 ± 22.1	42.9 ± 46.5	0.622
Ventilator (days)	7.7 ± 9.1	20.8 ± 54.7	0.722
CRRT (days)	15.8 ± 14.4	8.9 ± 5.6	0.514
Sepsis	17 (31.5)	26 (30.2)	0.989
Septic shock	28 (51.9)	27 (31.4)	0.045
Trace elements, μg/dL			
Zinc	48.6 ± 17.5	55.2 ± 20.3	0.054
Selenium	9.0 ± 2.4	10.0 ± 2.6	0.037
Copper	61.6 ± 12.8	103.1 ± 23.0	0.000
Manganese	10.5 ± 4.7	9.6 ± 3.8	0.213
Nutrition status, *n* (%)			0.925
Well nourished	17 (31.5)	30 (34.9)	
Malnourished	37 (68.5)	56 (65.1)	

*APACHE*, Acute Physiology and Chronic Health Evaluation; *SOFA*, Sequential Organ Failure Assessment; *SAPS*, Simplified Acute Physiology Score; *ICU*, intensive care unit; *CRRT*, continuous renal replacement therapy

**Fig. 2:**
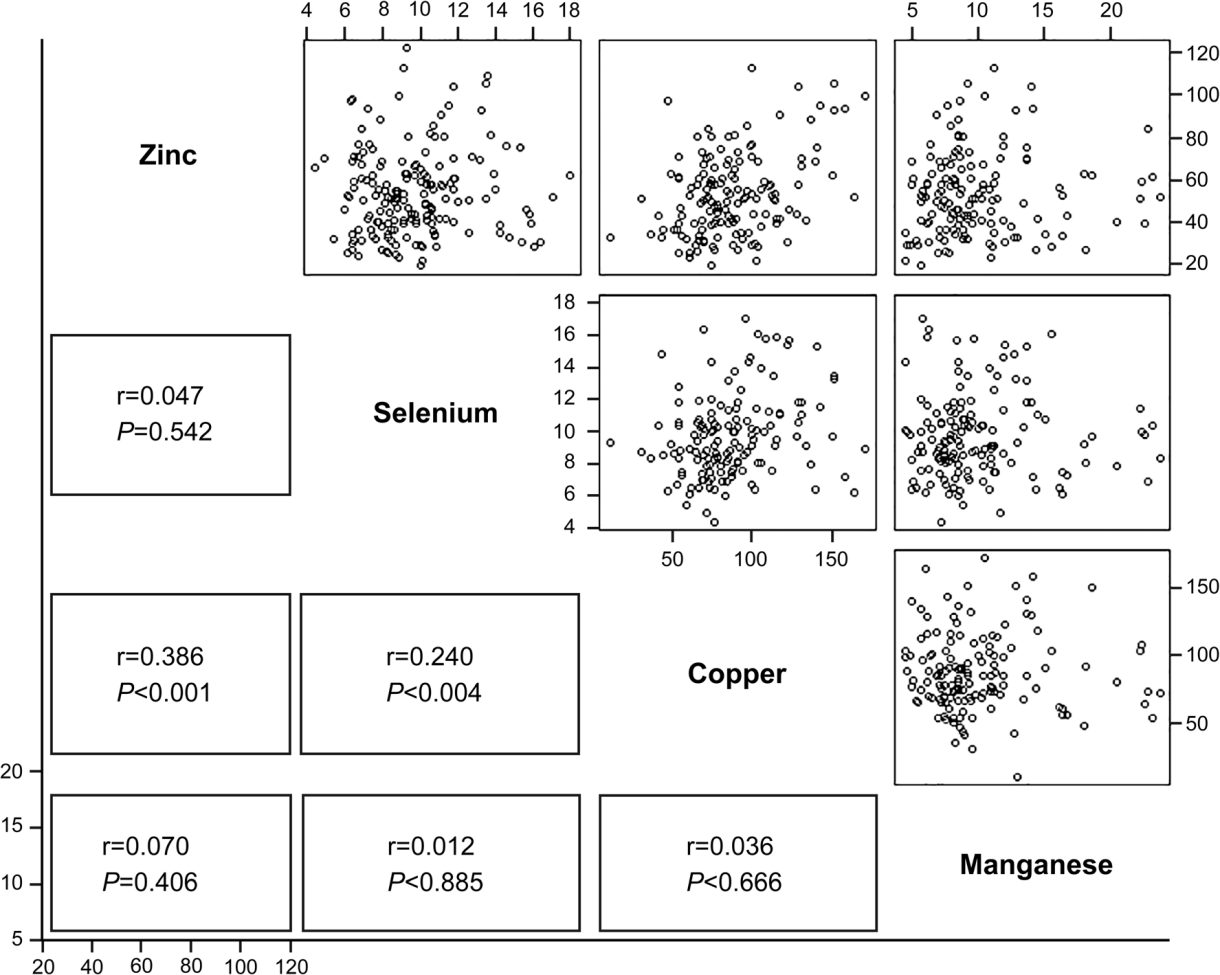
Correlations between serum trace element levels at intensive care unit (ICU) admission

**Fig. 3:**
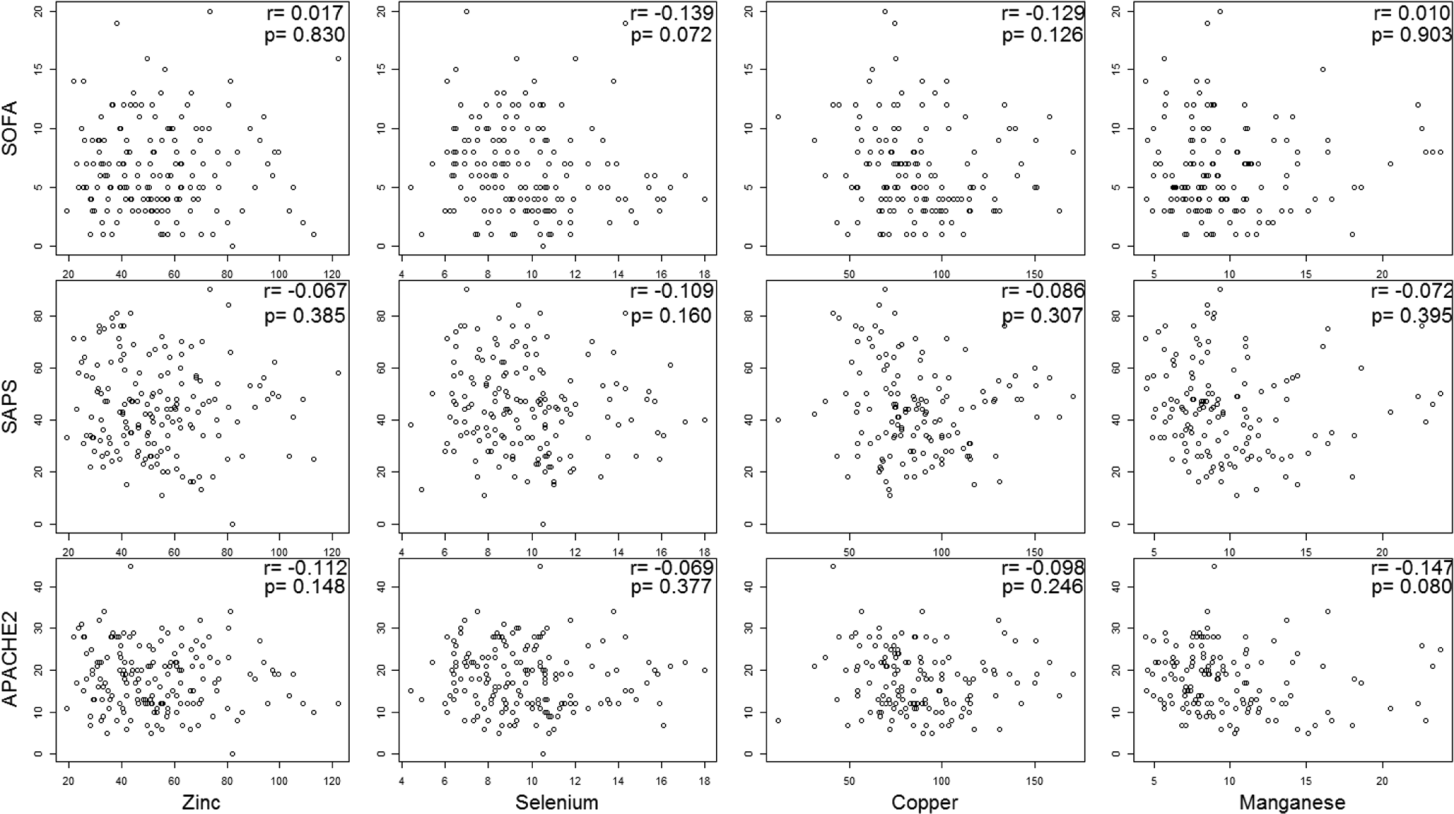
Correlations between severity scores and serum concentrations of trace elements. APACHE, Acute Physiology and Chronic Health Evaluation; SOFA, Sequential Organ Failure Assessment; SAPS, Simplified Acute Physiology Score

Follow-up of the serum trace element levels was conducted at 2-week intervals from the time of ICU admission for a maximum of 6 weeks (Table 1). There was no change in blood Se levels at the time of ICU admission. Thus, patients did not receive Se supplementation during the research period. During these 6 weeks, the serum Se concentration did not show a decreasing trend (Fig. 1). A matrix scatter plot analysis was performed to evaluate the correlation between trace element concentrations measured at the early stage of ICU admission. Positive correlations were evident between serum concentrations of Zn and Cu (*r* = 0.386, *P* < 0.001) and between Se and Cu (*r* = 0.24, *P* = 0.004) (Fig. 2). The correlations between other trace elements were not statistically significant (Fig. 2). Analysis of the correlations between severity scores and trace element concentrations revealed an inverse correlation between Se and SOFA score and between Mn and APACHE II score, although the differences were not statistically significant. Analysis of the correlations between other serum trace element concentrations and severity scores revealed no significant relationships (Fig. 3).

**Fig. 4:**
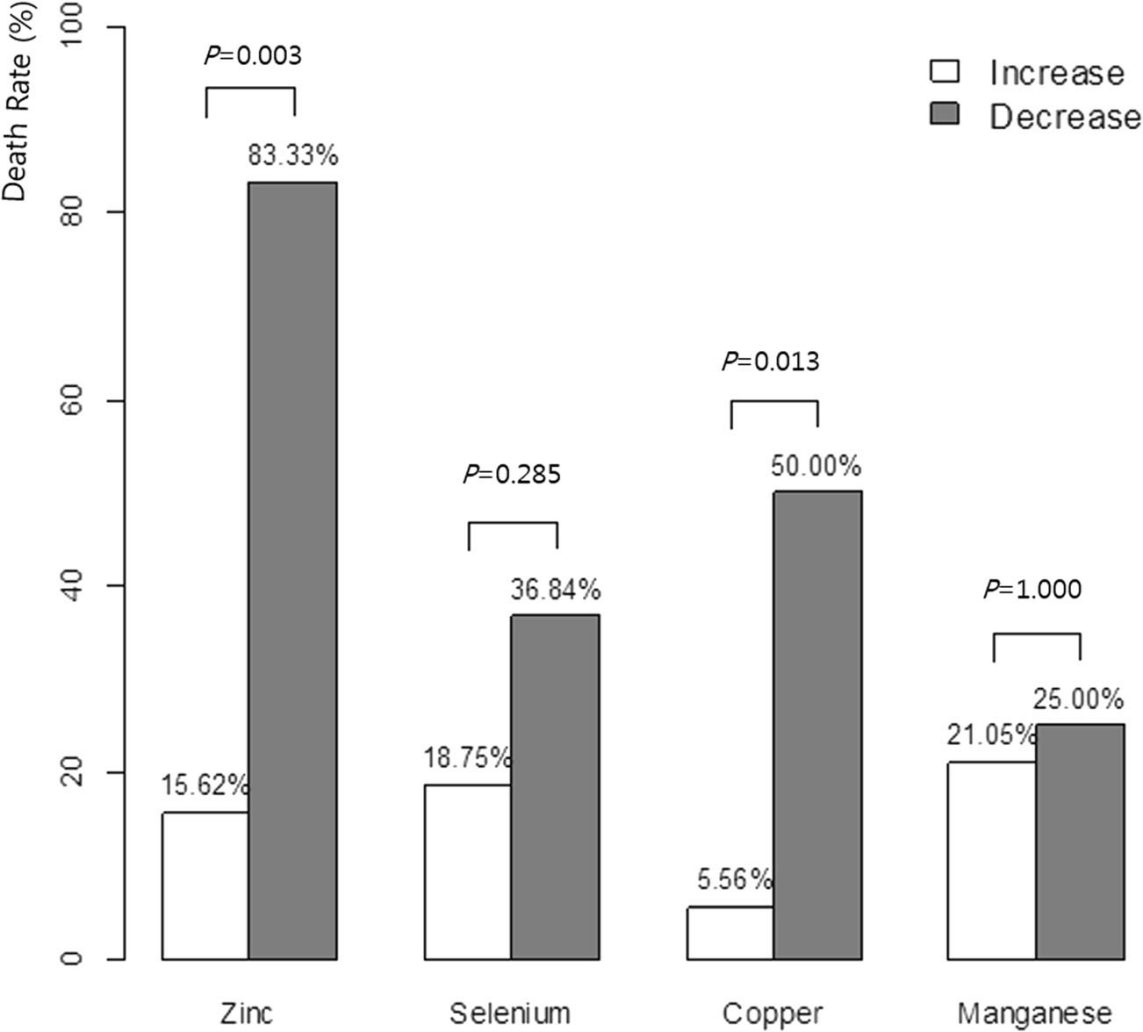
Comparisons of mortality according to the increase and decrease in serum trace element concentrations after 2 weeks of intensive care unit (ICU) admission

The relationships between mortality and serum trace element concentrations measured at the time of ICU admission and after 2 weeks were analyzed using Fisher’s exact tests (Fig. 4). Patients with decreased serum Zn concentration after 2 weeks showed a significantly higher mortality (mortality of patients with increased Zn vs. mortality of patients with decreased Zn: 15.6 vs. 83.3%, *P* = 0.003). In addition, patients with decreased serum Cu concentration after 2 weeks had a significantly higher mortality (mortality of patients with increased Cu vs. mortality of patients with decreased Cu: 5.6 vs. 50.0%, *P* = 0.013). Patients with decreased serum Se and Mn concentrations also tended to show a higher mortality, but the difference was not statistically significant (*P* = 0.285 and *P* = 1.000, respectively).

## Discussion

The ages of the subjects in the present study ranged from 18 to 90 years. In a serum trace element concentration study of a healthy group in Korea, the levels of trace elements were low in subjects older than 60 years. In the same study, the levels of Se, Cu, and Zn differed significantly among men and women [8]. However, in our study, there were no significant differences in the serum levels of trace elements according to age and sex (Tables 3 and 4). Several micronutrients, especially the serum concentrations of the trace elements associated with oxidation, are reportedly lower in septic patients [9]. Therefore, we believe there might be a difference between our study of critically ill patients and those involving healthy subjects.

Plasma zinc is a good indicator of zinc intake in the absence of systemic inflammation [10]. In the present study, low serum levels of Zn were observed in 76% of patients at the time of ICU admission. Our study involved a large number of patients with septic conditions (sepsis 82.6% and septic shock 43.7%). Our result is consistent with prior reports of low serum Zn level in 86–95% of critically ill patients [1, 2, 4, 6, 11].

Zn is a trace element associated with immune function and wound healing. Low serum levels of Zn are thought to influence the prognosis of ICU patients. In the present study, daily supplementation with Zn (0.5 mg/day) was provided through a standard trace element formulation. After 2 weeks, the average serum Zn concentration was higher than that at an early stage during ICU admission (53.4 to 72.8 μg/dL). The mortality rate was 15.6% for the group with increased Zn level and 83.3% for that with decreased Zn level. The group with decreased Zn level showed a significantly higher mortality rate. Young et al. [12] compared a group supplemented with 2.5 mg elemental Zn to a control group in a double-blinded randomized control trial comprising 68 mechanically ventilated patients with severe closed head injury. The group without Zn supplementation displayed a higher 30-day mortality than the group with Zn supplementation did, but the difference was not statistically significant (26 vs. 12%, *P* = 0.09). Heyland et al. [3] conducted a meta-analysis of four studies on the effects of Zn supplementation on clinical outcomes. Zn supplementation tended to decrease mortality (relative risk = 0.63, 95% confidence interval [CI] 0.25 to 1.59, *P* = 0.33) and ICU length of stay, without any statistical significance (− 0.35 days, 95% CI − 0.85 to − 0.15; *P* = 0.17). On the contrary, we observed a distinct mortality difference based on the change in serum Zn level during ICU stay, which may be attributable to patient diversity and the larger sample size in our study.

In our study, the group with normal Zn level had a significantly longer length of stay in hospital than the group with a low initial serum level of Zn did (38.9 vs. 52.8, *P* = 0.012). As all patients routinely received supplemented trace elements, we did not examine the difference between the supplementation and control groups. Another randomized control trial [13] involving 32 critically ill trauma patients admitted to a surgical ICU compared the outcomes of patients supplemented with 500 μg Se, 500 μg Se plus 150 mg α-tocopherol plus 13 mg Zn supplementation group, and a placebo group. No statistically significant difference was observed between the ICU length of stay and mortality in the three groups. Another randomized controlled trial of 20 patients with burn injuries examined the effects of Zn supplementation on the ICU period of stay. In this study, standard trace elements such as 1.3 mg Cu (20 μmol), 31.3 μg Se (0.4 μmol), and 6.5 mg Zn (100 μmol) were provided to the control group, while 2.6 mg Cu (40.4 μmol), 156 μg Se (2.0 μmol), and 26.5 mg Zn (406 μmol) were administered intravenously to the supplementation group, beginning on day 8 of ICU admission. There was no statistically significant difference between periods regarding the length of stay for the two groups [9]. However, after correcting for the burn area, the supplementation group had a significantly shorter length of stay compared to that of the control group (30 vs. 39 days, *P* = 0.034). In the present study, patients with normal Zn level had a longer length of stay. This may be associated with the shorter length of stay for patients with low serum levels of Zn who died during the study period (19.0 and 33.3% of patients died within 7 and 14 days, respectively), which affected the average length of stay in the group of patients with low serum levels of Zn.

Besecker et al. [4] examined serum Zn concentrations in 56 subjects, including 22 participants with sepsis, 22 critically ill control participants, and 12 healthy subjects. Compared to the healthy subjects, the critically ill control participants had lower Zn concentrations, and the concentrations were further decreased in participants with sepsis. The systemic inflammatory response syndrome (SIRS) group also displayed a significantly lower Zn concentration (*P* = 0.02). In addition, an inverse correlation was reported between the Zn level and SOFA score in patients with cardiovascular dysfunction. We expected similar results, but the sepsis and septic shock groups failed to show higher low Zn levels, and there was no correlation between Zn level at the time of ICU admission and severity score.

Many studies have focused on low serum levels of Se in ICU patients as compared to other trace elements. Recently, studies have used serum Se to identify Se deficiency; however, Se deficiency cannot be diagnosed by serum Se measurement alone because selective uptake of Se by body tissues result from the translocation of selenoenzymes due to increased vascular permeability. Therefore, serum Se levels may not represent the actual Se status [14]. However, it remains unclear whether low serum Se level directly impacts clinical outcomes, or whether it is secondary to a higher requirement for Se and protection against oxidative stress in more severe illnesses [10].

In one study, patients with SIRS displayed a 40% lower Se level than healthy patients [15]. In the present study, the average serum Se concentration measured at the time of ICU admission was 9.7 ± 2.6 (4.4–18.0) μg/dL, and only 1.8% of patients had Se levels below the normal range (5.8–23.4 μg/dL). The average Se concentrations measured on days 14, 28, and 42 without any Se supplementation were 8.9 (*n* = 35), 8.2 (*n* = 11), and 8.0 (*n* = 4) μg/dL, respectively, indicating no decreasing trend in serum Se concentration. The serum Se concentration was not significantly related to patient mortality (*P* = 0.821) and had no correlation with treatment outcomes (e.g., hospital and ICU length of stay).

Some studies have reported that Se supplements have a positive effect on the patient’s clinical outcome [6, 15–17], while others have reported no effect or borderline positive effects on respective outcome parameters [14, 18, 19]. Xavier et al. [15] analyzed the correlation between Se level and mortality in 31 ICU patients and reported that the survivors had higher serum Se levels. Fiona et al. [6] examined the correlations between Cu/Zn/Se levels and mortality/outcomes in 125 SIRS patients treated in the ICU. The mortality rate increased as the Se level decreased, but no statistical significance was observed (0.35, 95% CI 0.01–5.68 vs. 0.22, 95% CI 0.04–0.72, *P* = 0.43). Manzanares et al. [16] compared serum Se levels in 36 ICU patients and 23 normal participants and reported that the mortality rate increased as the Se level decreased. However, no statistical significance was observed due to the small number of participants (*P* = 0.074). In this study, the mortality rate tended to be higher among patients with lower Se levels after a 2-week follow-up, although the difference was not statistically significant (18.8 vs. 36.8%, *P* = 0.285).

Low Se level has been particularly related to sepsis [16, 20, 21] and is inversely correlated with the severity score [15, 21]. Manzanares et al. [16] reported that patients with SIRS and multiorgan dysfunction displayed decreased serum Se levels. Xavier et al. [16] reported that the Se level decreased as the APACHE score increased in sepsis. In 2013, Huang et al. [22] conducted a meta-analysis of Se supplementation studies and reported that Se supplementation decreased the mortality rate among sepsis patients. In the present study, patients with a serum Se concentration < 8.0 μg/dL (*n* = 48) were significantly more likely to be malnourished (*P* = 0.005) and to develop sepsis (*P* = 0.045). Of the severity indices for critically ill patients, the SOFA score was inversely correlated with serum Se concentration, although the difference was not statistically significant (*r* = − 0.139, *P* = 0.072). This result was similar to that reported by Mishra et al. [21], in which the serum Se concentration was inversely correlated with the SOFA score (*P* = 0.03). The difference in the present study might have been statistically significant had the number of participants been greater.

Although around 38% of patients in our study had low Cu levels, we cannot state that they had Cu deficiency. The subjects of our study were under physiological stress, a condition in which the body increases serum Cu levels due to increased synthesis of the protein ceruloplasmin, which contains most of the Cu in serum [10]. Thus, serum Cu levels in these subjects could simultaneously be pushed upward by increased ceruloplasmin synthesis and pushed downward by some degree of Cu deficiency. Generally, measurement of serum Cu and ceruloplasmin is the most common method of assessing Cu status, despite ceruloplasmin being a positive acute-phase reactant. Although we use this method to measure Cu levels in our ICU because serum analysis is the only available measurement method, it is unclear whether it reflects the state of the Cu level in the patients’ whole body.

Few studies have addressed Cu supplementation and the relevant outcomes. In the present study, patients with increased serum Cu concentration after 2 weeks of supplementation with standard trace elements had a mortality rate of 5.6%, while those patients with a decreased serum Cu concentration had a mortality rate of 50.0% (*P* = 0.013). Berger et al. [9] investigated a control group supplemented with Cu through a standard trace element formulation and those supplemented with twice the amount of standard trace elements. The supplementation group had significantly fewer 30-day infectious episodes and a significantly shorter length of stay after correcting for the area of the burn (30 vs. 39 days, *P* = 0.034). However, the results were not associated with Cu supplementation but with the increased amount of supplemented Se. Although there may appear a distinct mortality difference based on the change in serum Cu concentration, our study did not focus on single Cu supplementation.

In our study, Cu was the second-most deficient trace element after Zn. The serum Cu concentration was significantly correlated with serum Zn and Se concentrations (Fig. 2). Thus, low serum levels of Cu might be suspected in patients with low Zn or Se levels, necessitating Cu supplementation.

There are limited studies on Mn levels in critically ill patients and an increasing concern about the side effects associated with Mn accumulation rather than Mn deficiency [1]. However, in a small-scale study of normal participants, a group that ingested Mn-free meals over a long period showed serum Mn deficiency [22, 23]. However, we are uncertain that low serum Mn levels are definitely correlated with real Mn deficiency in the human body. In the present study, the proportion of patients with low serum Mn levels was very low (2.1%). In addition, no particular change was evident even with the daily supplementation of trace elements during the follow-up. There was no significant difference in the mortality rates between the groups with increased and decreased Mn concentrations during follow-up serum collection (Fig. 4). Thus, ordinary Mn supplementation seems unnecessary for patients with normal Mn levels. However, regular monitoring of Mn level is desirable for patients undergoing long-term ICU treatment, particularly those on total PN.

Our study has some limitations. First, many patients did not participate in the entire research process due to earlier ICU discharge or death. We were able to obtain only a few samples after the second week. Second, some patients received enteral nutrition as well as intravenous alimentation, depending on their progress after ICU admission; thus, the amount of trace elements supplied via enteral nutrition and their clinical effects could not be accurately determined. Third, we failed to measure ceruloplasmin activity, despite the significant decrease in Cu level observed at ICU admission. Fourth, we provided a daily injection of trace elements using a standard trace element formulation during the study period. The current Canadian guidelines and the recently published ASPEN guidelines do not recommend supplementation of micronutrient cocktails or trace elements as a single strategy in ICU patients [5, 24]. Owing to the lack of options for supplying trace elements, the use of a standard trace element formulation was the easiest and most economical means of trace element supplementation. The cost of intravenous standard trace element formulation for daily use in Korea is only $2. This cost is comparable to that of a single Zn-supplying formulation with the same amount of Zn (10 mg) that costs more than $20. Moreover, there is no available single Cu- and Mn-supplying formulation in Korea. Finally, we were unable to ascertain whether the subjects had received transfusion (fresh frozen plasma, packed red blood cell, platelet phoresies, cryoprecipitate, etc.). The effect of blood transfusions on serum trace elements was not considered.

The present study is relevant because the observations of long-term trace element levels involved a follow-up every 2 weeks during a total of 6 weeks (four measurements) in the ICU; however, many patients failed to complete the study owing to early ICU discharge or death. Nevertheless, we observed a high incidence of low serum Zn level and low serum Cu level at ICU admission. Patients with increased serum Zn and Cu concentrations during the ICU stay had significantly lower mortality. Serum Cu concentration showed a statistically significant correlation with serum Zn and Se concentrations.
